# Prevalence and *in vitro* antifungal susceptibility of commensal yeasts in the external ear canal of cats

**DOI:** 10.1186/s12917-021-02995-7

**Published:** 2021-08-28

**Authors:** Sara Niae, Chompoonek Yurayart, Naris Thengchaisri, Panpicha Sattasathuchana

**Affiliations:** 1 Graduate Student in Veterinary Clinical Studies, Faculty of Veterinary Medicine, Kasetsart University, 10900 Bangkok, Thailand; 2 Department of Microbiology and Immunology, Faculty of Veterinary Medicine, Kasetsart University, 10900 Bangkok, Thailand; 3 Department of Companion Animal Clinical Sciences, Faculty of Veterinary Medicine, Kasetsart University, 50 Ngamwongwan Rd, Latyao, Jatujak, 10900 Bangkok, Thailand

**Keywords:** Cat external ear canal, Commensal yeasts, *In vitro* antifungal susceptibility

## Abstract

**Background:**

Lifestyle factors such as hair length, the frequency of ear cleaning and bathing, age, cat rearing, and sex may contribute to opportunistic yeast infections in the external ear canal of cats. This study aimed to determine the prevalence of commensal yeast organisms in cats’ external ear canals, evaluate their predisposing lifestyle factors, and test the susceptibility of *Malassezia pachydermatis* to antifungal agents.

**Results:**

A total of 53 cats (33 male and 20 female) seronegative for feline leukemia virus and feline immunodeficiency virus were enrolled in this study. Their mean age (± standard deviation) was 6.04 (± 3.49) years. Fungal cultures and polymerase chain reaction tests were performed to identify the yeast species derived from the external ear canal. The association between lifestyle factors and the presence of *M. pachydermatis* was evaluated using Fisher’s exact test. The susceptibility of *M. pachydermatis* to antifungal agents was also analyzed. *M. pachydermatis* was the most frequently recovered yeast species, with a prevalence of 50.94 % (95 % confidence interval [CI]: 36.84–64.94 %). There was an association between hair length and a positive culture for *M. pachydermatis* (*p* = 0.0001). The odds of a negative culture for *M. pachydermatis* among short-haired cats was 11.67 (95 % CI, 3.22–42.24) times higher than that among long-haired cats (*p =* 0.0002). There was also an association between the frequency of ear cleaning and the presence of *M. pachydermatis* (*p* = 0.007). The odds of a negative culture for *M. pachydermatis* in cats that were receiving ear cleaning at intervals of ≤ 2 weeks was 5.78 (95 % CI, 1.67–19.94) times greater than that of cats receiving ear cleaning at intervals greater than 2 weeks or never (*p* = 0.0055). Ranges of minimum inhibitory concentrations (MICs) and minimum fungicidal concentrations for itraconazole, ketoconazole, miconazole, and terbinafine against *M. pachydermatis* were ≤ 0.063–4 and ≤ 0.063–≥32, ≤ 0.063–8 and 0.125–≥32, ≤ 0.063–≥32 and 0.5–≥32, and ≤ 0.016–1 and 0.125–8 µg/ml, respectively.

**Conclusions:**

*M. pachydermatis* was the most commonly identified yeast organism in the external ear canal of healthy cats. Hair length and the frequency of ear cleaning played a role in the colonization of *M. pachydermatis*. The *M. pachydermatis* isolates had various MIC levels for common fungicides.

## Background

*Malassezia* spp. is a commensal yeast found on the mucosa and skin of mammals. Overgrowth of this organism may cause a local or systemic disease in humans and cats [1, 2]. *Malassezia pachydermatis* is a nonlipid-dependent yeast species, and it is the most commonly isolated yeast organism from the ear canal of cats [3–6]. The prevalence of *M. pachydermatis* in cats with otitis externa and cats without otitis externa ranges from 24–60 % and 17.6–23 %, respectively [3–6]. Although the prevalence of *M. pachydermatis* in the ear canal of cats has been widely reported around the world [7], studies on the diversity of commensal yeast organisms in healthy cats have been limited. Hair length is a major contributing factor to dermatophytosis in healthy cats [8]. Therefore, lifestyle factors such as hair length, as well as other factors such as season or climate, may contribute to the risk of yeast overgrowth in cats’ ears [8, 9].

Topical or systemic azole antifungal agents have been widely used in cats with otitis externa and dermatitis associated with *Malassezia* yeast infections [10, 11]. Several reports of antifungal agent resistance have been recorded due to the inappropriate use of antifungal agents and organisms’ genetic mutations [12–15]. Therapeutic failure against *M. pachydermatis* infection in dogs has been reported due to the use of multiple antifungal drugs [12, 13]. However, few researchers have investigated the levels of antifungal susceptibility of yeasts in cats [7]. The aims of this study were to (1) determine the diversity of commensal yeasts in the external aural canal of cats seronegative for feline leukemia virus (FeLV) and feline immunodeficiency virus (FIV), (2) determine lifestyle factors that predispose cats to *M. pachydermatis*, and (3) evaluate the *in vitro* antifungal susceptibility of *M. pachydermatis* colonizing the external ear canal of cats.

## Results

All 53 cats seronegative for FeLV and FIV infection appeared to be healthy, with no history of ear canal or ear pinna diseases and no history of pruritus. All enrolled cats had no abnormal clinical findings of the external ear canal (a very slight amount of cerumen, no visible color of the cerumen, no ear canal stenosis, no ear pinna erythema, and no otopedal reflex). The demographic characteristics of the cats, including age (mean ± standard deviation [SD]: 6.04 ± 3.49 years), sex (male: 62.26 %, female: 37.74 %), body weight (mean ± SD): 5.08 ± 1.48 kg), breed (Domestic Short-Haired: 45.28 %, Bengal: 3.77 %, Persian: 33.96 %, Maine Coon: 7.55, Mixed Long-Haired: 9.44 %), and cat rearing (indoor only: 90.57 %, outdoor access: 9.43 %), are shown in Table 1.

**Table 1:** Demographic characteristics

Characteristic	All cats
Number	53
Age (years)	
Mean$($±standard deviation)	6.04 (± 3.49)
Sex (%; n)	
Male	62.26 (33)
Female	37.74 (20)
Body weight (kg)	
Mean (± standard deviation)	5.08 (± 1.48)
Breed (%; n)	
Domestic Short-Haired	45.28 (24)
Bengal	3.77 (2)
Persian	33.96 (18)
Maine Coon	7.55 (4)
Mixed Long-Haired(hair shaft length > 3 cm)	9.44 (5)
Cat rearing (%; n)	
Indoor only	90.57 (48)
Outdoor access	9.43 (5)

In the present study, 10 yeast species were identified from the external ear canal of cats: *M. pachydermatis*, *Malassezia nana*, *Malassezia cuniculi, Malassezia furfur, Candida ciferrii*, and other yeast species (*Aureobasidium melanogenum, Cystobasidium calyptogenae, Moesziomyces aphidis, Rhodotorula mucilaginosa*, and *Sympodiomycopsis* sp.). The prevalence (95 % confidence interval [CI]) of *M. pachydermatis* was 50.94 % (36.84–64.94 %; Table 2). The prevalence (95 % CI) of *M. nana, M. cuniculi*, and *M. furfur* was 16.98 % (8.07–29.80), 1.89 % (0.05–10.07 %), and 1.89 % (0.05–10.07 %), respectively. The prevalence (95 % CI) of *C. ciferrii* was 9.42 % (3.13–20.66 %). The prevalence (95 % CI) of each of the remaining yeasts (*Aureobasidium melanogenum, Cystobasidium calyptogenae, Moesziomyces aphidis, Rhodotorula mucilaginosa*, and *Sympodiomycopsis* sp.) was 1.89 % (0.05–10.07 %).

**Table 2:** Prevalence (95 % confidence interval) of 10 identified yeast species

Identified yeast genus	Number of cats		Prevalence (%; 95 % confidence interval)
	**Negative culture**	**Positive culture**	
*Malassezia pachydermatis*	26	27	50.94 (36.84–64.94)
*Malassezia nana*	44	9	16.98 (8.07–29.80)
*Malassezia cuniculi*	52	1	1.89 (0.05–10.07)
*Malassezia furfur*	52	1	1.89 (0.05–10.07)
*Candida ciferrii*	48	5	9.42 (3.13–20.66)
*Aureobasidium melanogenum*	52	1	1.89 (0.05–10.07)
*Cystobasidium calyptogenae*	52	1	1.89 (0.05–10.07)
*Moesziomyces aphidis*	52	1	1.89 (0.05–10.07)
*Rhodotorula mucilaginosa*	52	1	1.89 (0.05–10.07)
*Sympodiomycopsis* sp.	52	1	1.89 (0.05–10.07)

The prevalence (95 % CI) of *M. pachydermatis* among short-haired and long-haired cats was 11.32 % (4.27–23.23 %) and 39.62 % (26.45–54.0 %), respectively (Table 3). There was an association between hair length and a positive culture for *M. pachydermatis* (*p* = 0.0001). The odds (95 % CI) of a negative culture for *M. pachydermatis* among short-haired cats was 11.67 (3.22–42.24) times higher than that among long-haired cats (*p =* 0.0002).

**Table 3:** Lifestyle factors of cats with a positive culture of *Malassezia pachydermatis*

Lifestyle factors	Number of cats		*p*-value
	**Negative culture**	**Positive culture**	
**Hair length**			0.0001
Short-haired (≤ 3 cm)	20	6	
Long-haired (> 3 cm)	6	21	
**Ear cleaning frequency**			0.007
≤2 weeks	12	6	
>2 weeks or never	9	26	
**Age group**			0.7846
≤6 years	15	14	
>6 years	11	13	
**Bathing frequency**			0.0537
≤1 month	3	10	
>1 month or never	23	17	
**Cat rearing**			0.1917
Indoor only	22	26	
Outdoor access	4	1	
**Sex**			0.7786
Male	17	16	
Female	9	11	

The prevalence (95 % CI) of *M. pachydermatis* in cats that had been receiving ear cleaning at intervals of ≤ 2 weeks and > 2 weeks or never was 11.32 % (4.27–23.03 %) and 49.06 % (35.06–63.16 %), respectively. There was an association between ear cleaning frequency and the presence of *M. pachydermatis* (*p* = 0.007). The odds (95 % CI) of a negative culture for *M. pachydermatis* in cats that were receiving ear cleaning at ≤ 2-week intervals was 5.78 (1.67–19.94) times higher than among cats receiving ear cleaning at > 2-week intervals or never (*p* = 0.0055).

The prevalence (95 % CI) of *M. pachydermatis* in cats aged ≤ 6 years and > 6 years was 26.42 % (15.26–40.33 %) and 24.53 % (13.75–38.28 %), respectively. The prevalence (95 % CI) of *M. pachydermatis* in cats that were bathed at intervals of ≤ 1 month and > 1 month or never was 18.87 % (9.44–31.97 %) and 32.08 % (19.92–46.32 %), respectively. The prevalence (95 % CI) of *M. pachydermatis* in indoor-only cats and cats with outdoor access was 49.06 % (35.06–63.16 %) and 1.89 % (0.05–10.07 %), respectively. The prevalence (95 % CI) of *M. pachydermatis* in male cats and female cats was 30.19 % (18.34–44.34 %) and 20.75 % (10.84–34.11 %), respectively. There was no association between a positive culture of *M. pachydermatis* and lifestyle factors such as age (*p* = 0.7846), frequency of bathing (*p* = 0.0537), cat rearing (*p* = 0.1917), and sex (*p* = 0.7786) (Table 3).

The results of the antifungal susceptibility testing for the most common yeast, *M. pachydermatis*, from 27 cats are shown in Table 4. Overall, 41 isolates of *M. pachydermatis* from these cats were analyzed for antifungal susceptibility. The ranges of the minimum inhibitory concentrations (MICs) for itraconazole (ITZ), ketoconazole (KZ), miconazole (MZ), and terbinafine (TERB) against all isolates of *M. pachydermatis* were ≤ 0.063–4, ≤ 0.063–8, ≤ 0.063–≥32, and ≤ 0.016–1 µg/ml, respectively. The ranges of the minimum fungicidal concentrations (MFCs) for ITZ, KZ, MZ, and TERB against all isolates of *M. pachydermatis* were ≤ 0.063–≥32, 0.125–≥32, 0.5–≥32, and 0.125–8 µg/ml, respectively.

**Table 4:** Susceptibility testing for 41 isolates of *Malassezia pachydermatis*

Itraconazole		Ketoconazole		Miconazole		Terbinafine	
**MIC (n)**	**MFC (n)**	**MIC (n)**	**MFC (n)**	**MIC (n)**	**MFC (n)**	**MIC (n)**	**MFC (n)**
4 (1)	≥ 32 (1)	8 (1)	≥ 32 (2)	≥ 32 (10)	≥ 32 (9)	1 (2)	8 (4)
1 (2)	16 (1)	2 (2)	16 (1)	16 (1)	16 (13)	0.5 (5)	4 (19)
0.5 (1)	8 (2)	1 (5)	8 (3)	8 (3)	8 (14)	0.25 (9)	2 (12)
0.25 (4)	1 (1)	0.25 (15)	4 (3)	4 (4)	4 (2)	0.125 (9)	1 (5)
0.125 (1)	0.5 (5)	0.125 (9)	2 (4)	2 (13)	2 (1)	0.063 (8)	0.125 (1)
0.063 (32)	0.25 (2)	0.063 (9)	1 (7)	1 (1)	1 (1)	0.031 (2)	
	0.125 (11)		0.5 (19)	0.5 (5)	0.5 (1)	0.016 (6)	
	0.063 (18)		0.25 (1)	0.125 (1)			
			0.125 (1)	0.063 (3)			

*MIC* minimum inhibitory concentration (µg/ml); *N* represents the number of isolates that presented the indicated MIC or MFC; *MFC* minimum fungicidal concentration (µg/ml)

## Discussion

In this study, the identified organisms from the external ear canals of 53 cats were *Malassezia pachydermatis*, *Malassezia nana*, *Malassezia cuniculi, Malassezia furfur, Candida ciferrii, Aureobasidium melanogenum, Cystobasidium calyptogenae, Moesziomyces aphidis, Rhodotorula mucilaginosa*, and *Sympodiomycopsis* sp. The most common positive cultured yeast was *M. pachydermatis*, with a prevalence (95 % CI) of 50.94 % (36.84–64.94 %). Because of its high abundance, *M. pachydermatis* was selected in this study of risk factors and susceptibility testing. Lifestyle factors such as hair length and the frequency of ear cleaning contributed to the risk of having a positive culture for *M. pachydermatis* from the cats’ external ear canals. This study is the first report of the MICs and MFCs of ITZ, KZ, MZ, and TERB against *M. pachydermatis* in cats.

All 53 enrolled cats were apparently healthy, as confirmed by taking their history and conducting a general physical examination, complete blood count testing, blood chemistry testing, and serology testing for FeLV and FIV infection. All positive cultured organisms from the external ear canal of the cats were considered commensal organisms. The cat’s cerumen was initially cultured on various cultured media to increase the variety of isolated yeast organisms followed by polymerase chain reaction (PCR) and DNA sequencing. Conventional yeast identification using culture media is often time-consuming, and it is difficult to differentiate lipid-dependent yeast species (*M. nana*, *M. cuniculi*, and *M. furfur*). Molecular identification of yeast species is a quick method to confirm yeast species.

The *Malassezia* species identified in this study were *M. pachydermatis*, *M. nana*, *M. cuniculi*, and *M. furfur.* These organisms are commensal yeasts of the ear canal and skin in felids and other domestic animals [3, 5, 9, 16]. The prevalence of *M. pachydermatis* and *M. nana* has previously been reported in cats without otitis externa to be 13.4–41.1 and 3 %, respectively [6, 9, 16–18]. The prevalence of *M. pachydermatis* (40.9 %) and *M. nana* (16.98 %) in healthy cats from this study was higher than that in previous reports. This discrepancy may be explained by the methods of sample collection and geographic differential effects. Previous studies performed sample collection on the unilateral ear canal [9] and used an antiseptic solution on the ear pinna and canal surface before sample collection [16], whereas samples from this study were collected from both ear canals and did not use any antiseptics on the ear pinna or ear canal surface before sample collection. This may have resulted in an increased number of yeast organisms identified. Moreover, PCR tests and DNA sequencing were also performed in this study to identify the yeast species. Therefore, the prevalence of yeast organisms in this study may truly represent their prevalence better than other studies that used only macroscopic, microscopic, and biochemistry property identification [9, 16]. In addition, this study was performed in a tropical area. Geographical variation may play an important role in the prevalence of *M. pachydermatis* in this study. Moreover, the presence of *M. nana* has been primarily noted to be in cat external ear canals compared to other body parts, suggesting that this organism is adapted to this anatomical site [18].

Furthermore, this study was the first to report the prevalence of *M. cuniculi*, *M. furfur*, *C. ciferri, Aureobasidium melanogenum, Cystobasidium calyptogenae, Moesziomyces aphidis, Rhodotorula mucilaginosa*, and *Sympodiomycopsis* sp. in the external ear canal of cats. The genus *Candida* is an opportunistic pathogen that causes candidiasis and candidaemia in both humans and animals [19]. *Aureobasidium melanogenum* and *Moesziomyces* species are opportunistic pathogenic yeasts that cause fungemia [20–22] and trigger hypersensitivity pneumonitis in humans [23]. *Rhodotorula* spp. is an emergent opportunistic pathogen, particularly in immunocompromised humans [24, 25]. However, there have been no reports of systemic infection with these pathogens in cats. Additional studies should be performed to identify the pathogenesis of these opportunistic yeast organisms in cats. Moreover, more awareness and additional studies are needed about commensal yeast pathogen transmission from cats to humans within the same household. *Cystobasidium calyptogenae* and *Sympodiomycopsis* sp. have been reported as environmental yeast organisms in water and soil [26].

It should be noted that the yeasts isolated in this study were present on the skin of the ear canals of healthy cats. The pathogenic roles of these isolated organisms may be related to the host immune response as well as to yeast-specific virulence factors [2, 27]. Cats with FeLV, FIV, and glucocorticoid treatment are predisposed to yeast overgrowth [11, 28]. Cats with hypersensitivity to yeasts or their metabolites are likely to develop severe skin lesions [2]. Structural malformation of the ear canal, excessive sebum production, and otitis are associated with bacterial and yeast overgrowth [2, 27].

This study investigated the association between the presence of *M. pachydermatis* in the external ear canal of cats and lifestyle factors. Age, bathing frequency, cat rearing, and sex are not commonly considered to be predisposing factors for *M. pachydermatis* in cats; however, this study confirmed that hair length and the frequency of ear cleaning contribute to the risk of a positive culture for *M. pachydermatis* from cats’ external ear canals. This finding may be explained by the anatomy associated with hair length. For example, long-haired cats have hairs in the ear canal, which may increase its local humidity and the chance of yeast accumulation in the ear canal. Humidity and the presence of lipids and sebum may predispose cats to yeast overgrowth, leading to inflammation and the development of pathologic lesions [2].

The MICs of ITZ, KZ, MZ, and TERB against *M. pachydermatis* were evaluated using results modified from a previous report [29–31]. The MIC of ITZ against most isolates of *M. pachydermatis* in our study was 0.063 µg/ml (78.05 %, 32/41 isolated), which was similar to a previous report of antifungal susceptibility levels of ITZ against *M. pachydermatis* isolated from otitis and dermatitis dogs and cats [29, 31]. The highest MIC of ITZ was 4 µg/ml, which was consistent with a previous report of high MIC levels of ITZ (4–8 µg/ml) from planktonic cells of *M. pachydermatis* isolated from the skin and scales of dogs [30]. The MICs of KZ at 0.125–0.25 µg/ml observed in 59 % of *M. pachydermatis* isolates in our study were consistent with previous reports [30, 31]. The highest MIC of KZ at 8 µg/ml from this study was consistent with previous findings [30, 31].

Most isolates of *M. pachydermatis* (76 %) showed the same range of MICs for MZ, with many reports at 0.063-16 µg/ml [12, 31–35]. Interestingly, a high MIC of MZ at ≥ 32 µg/ml was detected in 24 % of our isolates. To our knowledge, the MIC of MZ at ≥ 32 µg/ml was the highest MIC level that has been reported to inhibit the growth of *M. pachydermatis* from cats. This could be due to antifungal resistance caused by inappropriate uses of MZ, which is commonly included in ear treatment solutions [12].

The MICs of TERB against *M. pachydermatis* were 0.016-1 µg/ml, similar to previous reports [29–31, 33, 36, 37]. The sensitivity to TERB may be explained by the limited use of TERB in Thailand to treat animals. Overall, the antifungal susceptibility of all tested drugs against *M. pachydermatis* from the external ear canal of healthy cats was comparable to most studies in animals. Interpretative breakpoints are not available for *Malassezia* yeasts; nonetheless, high MIC values were recognized for the isolates. Further studies of antifungal susceptibility testing obtained from clinically related isolates and reports of treatment failure are necessary.

## Conclusions

The present study revealed that *M. pachydermatis* was the most commonly isolated yeast organism from cats’ external ear canals. Hair length and frequency of ear cleaning played a role in the presence of *M. pachydermatis*. All *M. pachydermatis* isolates had various MIC levels against common fungicides.

## Methods

### Animals

A prospective cross-sectional study was performed at Kasetsart University Veterinary Teaching Hospital, Faculty of Veterinary Medicine, Bangkok, Thailand, from March to December 2020. Fifty-three healthy cats were enrolled in this study. None of the cats had an illness or required any kind of medical treatment for at least 3 months prior to the study. The age (≤ 6 years and > 6 years), sex (male and female), breed, and body weight of each cat were recorded. In addition, the lifestyle factors of the individual cats were assessed, including their hair length (short-haired and long-haired), frequency of ear cleaning (≤ 2 weeks and > 2 weeks or never), frequency of bathing (≤ 1 month and > 1 month or never), and history of cat rearing (indoor only and outdoor access). A general physical examination, complete blood count, and blood chemistry testing (blood urea nitrogen, creatinine, serum glutamate pyruvate transaminase, total protein, and albumin) were performed on all cats. A commercially available FeLV-FIV rapid immunochromatography test assay (Witness®, Lyon Cedex, France) was used to determine FeLV and FIV infection.

### Dermatological examination and specimen collection

Bilateral otoscopic examination of the ear pinnae and ear canal was performed on all of the cats. The appearance of the ear pinnae and the otopedal reflex were characterized and recorded according to the criteria of the clinical findings of the external ear canal, including the history of pruritus (absent or present), amount of cerumen (very slight, slight, or moderate to severe cerumen), the color of the cerumen (none visible, white, or yellow), ear canal stenosis (absent or present), erythematous (absent or present), and the otopedal reflex (absence or present) [38, 39]. Sterile cotton swabs were used for external ear canal sample collection for yeast isolation.

### Yeast isolation and phenotypic identification

An external ear canal swab was inoculated on culture media consisting of Sabouraud dextrose agar (SDA) (Difco, USA), Leeming and Notman agar (LNA) [40], and mycobiotic agar (Acumedia, MI, USA). Oxytetracycline (50 mg/L; Oxycline, Bangkok, Thailand) was added to all culture media to inhibit bacterial growth and allow the commensal yeasts to grow and easily be isolated. All cultured plates were incubated at 30 °C and examined daily for colony growth for 14 days. The growth rate, colony morphology, and microscopic features of the yeasts on each agar type were observed. To presumptively identify the yeast genus, a urea hydrolysis test was performed on all isolates.

All isolates of *Malassezia* yeasts were presumptively identified to the species level according to their lipid dependency and utilization from various lipid tests, including growth on lipid-free medium SDA, the utilization of castor oil (Cremophor EL, EL slant; Sigma-Aldrich, MO, USA), utilization of Tween 60-esculin agar (TE slant), and a Tween assimilation test [40].

### DNA extraction, PCR amplification, and DNA sequencing

DNA extraction was modified from a previous report [41]. In brief, lysis buffer solution comprised of sodium dodecyl sulfate salt (0.5 g; Himedia, Mumbai, India), NaCl (1.4 g; Carlo Erba, MI, USA), EDTA (0.73 g; Biobasic, Ontario, Canada), 1 M Tris-HCl (20 ml; Biobasic, Ontario, Canada) and 2-mercaptoenol (5 µl; Sigma-Aldrich, Steinheim, Germany) was added to the freeze-harvested yeast cells and they were incubated at 65 °C for 1 h (vortexed at least once during incubation). The lysate was purified using 500 µl of phenol:chloroform:isoamyl alcohol solution (vol:vol:vol of 25:24:1). The yeast suspension was thoroughly mixed by vortexing for 2 min to obtain a homogeneous suspension and then centrifuged at 14,000 rpm for 15 min. The clear upper aqueous phase was transferred to a new tube, an equal volume of cold isopropanol was added, and the tube was kept at − 20 °C overnight. The DNA was precipitated by centrifugation at 14,000 rpm and 4 °C for 20 min. Then, the DNA pellet was washed with 500 µl of 70 % ethanol and air-dried, dissolved in molecular-grade water, measured for DNA concentration using a NanoDrop (Thermo Scientific, MA, USA), and stored at − 20 °C until use.

The internal transcribed spacer (ITS) region of the ribosomal DNA gene of all DNA samples was amplified using the primer set SR6R (5′-AAGTATAAGTCGTAACAAGG-3′) and ITS4 (5′-TCCTCCGCTTATTGATATGC-3′) [42]. The PCR amplicons were visualized by 1.5 % agarose gel (Serva, Heidelberg, Germany) electrophoresis; all PCR products were purified and sequenced in both the forward and reverse directions using ABI BigDye Terminator v.3.1 (Macrogen Inc., South Korea).

The sequences were assembled bidirectionally and manually corrected for consensus sequences using BioEdit v.7.2.5 (https://bioedit.software.informer.com). Sequence similarity was searched using the BLASTn tool of the National Center for Biotechnology Information (http://blast.ncbi.nlm.nih.gov/Blast.cgi). The species identity of each yeast isolate was determined based on the sequence similarity of the ITS sequence. DNA alignment and phylogenetic analysis were performed using Molecular Evolution Genetics Analysis (MEGA X version 10, Pen State University, PA, USA). Gene sequencing of all representative isolates was submitted to GenBank (accession numbers: MW793487, MW815503, MW815569, MW816105, MW816147, MW816540, MW816624, MW816629, MW816824, MW816825, MW816915, MW816924, MW817021, MW817088, MW819644. MW819645, MW819646, MW819853, MW819854, MW824435, MW824436, MW824437, MW824438, MW824439, MW824440, MW824441, MW824442, MW824443, MW824444, MW824445, MW824446, MW824447, MW824448, and MW824449).

### Antifungal susceptibility testing

Antifungal susceptibility testing of *M. pachydermatis*, the most common isolated yeast species in this study, was performed in duplicate following the Clinical and Laboratory Standards Institute broth microdilution method (CLSI M27-A3) [43], using a modified version of the method described by Alvarez-Perez et al. [44]. Sabouraud dextrose broth (SDB) supplemented with 1 % Tween 80 (Sigma-Aldrich, MO, USA) was used as a diluting and culture medium in all steps of the antifungal susceptibility testing. The susceptibility of *M. pachydermatis* to the following antifungal agents was tested: ITZ (Sigma-Aldrich, MO, USA), KZ (Sigma-Aldrich, USA), MZ (Sigma-Aldrich, MO, USA), and TERB (Sigma-Aldrich, MO, USA). The final concentrations of the antifungal agents ITZ, KZ, and MZ were prepared at 10 different concentrations (0.063, 0.125, 0.25, 0.5, 1, 2, 4, 8, 16, and 32 µg/ml). TERB was prepared in concentrations of 0.016, 0.031. 0.063, 0.125, 0.25, 0.5, 1, 2, 4, and 8 µg/ml. The broth microdilution method was performed in 96-well microtiter plates (Sigma-Aldrich, MO, USA). The *M. pachydermatis* inoculum size was measured by spectrophotometry at 530 nm and adjusted with culture medium to obtain a final cell concentration of 5 × 10^3^–1 × 10^4^ CFU/ml. *Candida parapsilosis* ATCC 22,019 was included as the quality control isolate. After incubating at 30 °C for 72 h, MIC was determined by naked-eye visualization, and MFC was determined after 72 h of incubation by transferring 200 µl of suspension from all wells showing no visible growth to newly fresh SDB with 1 % Tween 80. The tubes were incubated at 30 °C, and the MFC was defined as the lowest concentration showing complete growth inhibition as demonstrated by the clear subculture tubes.

### Statistical methods

Significance was set at *p* < 0.05. The prevalence and 95 % CI of each yeast organism in the external aural canal in long-haired and short-haired cats were determined. A normality test was performed for all data using the Shapiro-Wilk test. Fisher’s exact test was used to analyze the association between lifestyle factors and the presence of *M. pachydermatis.*

## Data Availability

The data used and/or analyzed in the present study are available in the GenBank repository, accession numbers (MW793487, MW815503, MW815569, MW816105, MW816147, MW816540, MW816624, MW816629, MW816824, MW816825, MW816915, MW816924, MW817021, MW817088, MW819644. MW819645, MW819646, MW819853, MW819854, MW824435, MW824436, MW824437, MW824438, MW824439, MW824440, MW824441, MW824442, MW824443, MW824444, MW824445, MW824446, MW824447, MW824448, and MW824449). The phylogenetic data and the alignments have been stored in TreeBASE with a submission ID of 28,179 (accession URL: http://purl.org/phylo/treebase/phylows/study/TB2:S28179).

## Abbreviations

CI: Confidence interval
FeLV: Feline leukemia virus
FIV: Feline immunodeficiency virus
ITS: Internal transcribed spacer
ITZ: Itraconazole
KZ: Ketoconazole
LNA: Leeming and Notman agar
MFC: Minimum fungicidal concentration
MIC: Minimum inhibitory concentration
MZ: Miconazole
PCR: Polymerase chain reaction
SD: Standard deviation
SDA: Sabouraud dextrose agar
SDB: Sabouraud dextrose broth
TERB: Terbinafine
